# Identification of a Six-Gene Prognostic Signature Characterized by Tumor Microenvironment Immune Profiles in Clear Cell Renal Cell Carcinoma

**DOI:** 10.3389/fgene.2021.722421

**Published:** 2021-11-18

**Authors:** Lu Zhang, Jianlong Li, Mengzhao Zhang, Lu Wang, Tao Yang, Qiuya Shao, Xiao Liang, Minghai Ma, Nan Zhang, Minxuan Jing, Rundong Song, Jinhai Fan

**Affiliations:** ^1^ Department of Urology, the First Affiliated Hospital of Xi’an Jiaotong University, Xi’an, China; ^2^ Department of Urology, Xi’an NO.3 Hospital, the Affiliated Hospital of Northwest University, Xi’an, China; ^3^ Oncology Research Lab, Key Laboratory of Environment and Genes Related to Diseases, Ministry of Education, Xi’an, China

**Keywords:** gene signature, tumor microenvironment, tumor-infiltrating immune cells, immunotherapy, clear cell renal cell carcinoma

## Abstract

Clear cell renal cell carcinoma (ccRCC) is widely acknowledged to be extremely sensitive to immunotherapy, emphasizing the tremendous impacts on which the tumor microenvironment (TME) has shown. However, the molecular subgroups characterized by the TME features scarcely serve as the risk stratification guides in clinical practice for survival outcomes and immunotherapy response prediction. This study generated fresh insights into a novel TME-related prognostic signature derived from The Cancer Genome Atlas database using integrated bioinformatics analyses. Subsequently, Kaplan–Meier survival analysis, receiver operating characteristic analysis, and univariate and multivariate Cox regression analysis were performed to evaluate and validate the efficacy and the accuracy of the signature in ccRCC prognosis. Furthermore, we discovered that the risk score presented an increased likelihood of correlation with miscellaneous clinicopathological characteristics, natural killer cell-mediated cytotoxicity, immune cell infiltration levels, and immune checkpoint expression. These findings highlighted the notion that the six-gene signature characterized by the TME features may have implications on the risk stratification for personalized and precise immunotherapeutic management.

## Introduction

Clear cell renal cell carcinoma (ccRCC) is the most common subtype of renal cancer, making up nearly 70% of the diagnosed individuals (Jonasch et al., 2021; Jonasch et al., 2014; American Cancer Society, 2013). According to the latest guidelines, for patients in the early stage, partial nephrectomy is widely accepted as a preferred approach for a good prognosis (Ljungberg et al., 2015). However, there is no such comfort for patients with metastatic ccRCC, attributing to a striking 11.6% 5-year survival rate compared to 92.5% in patients with early-stage ccRCC (Howlader et al., 2017). Several targeted agents combined with immune checkpoint inhibitors are currently used as the optimal first-line therapy for ccRCC patients (Atkins and Tannir, 2018; Gill et al., 2018; Grimm et al., 2020), which show underestimated effects on distant metastasis control. To date, accumulating studies have shifted their efforts to proposing novel gene signatures or biomarkers that might become the potential tumor-specific targets of ccRCC (Sanchez and Simon, 2018; Ghatalia et al., 2019; Zhang et al., 2019). However, there are few predictive and robust biomarker guides in the first-line therapy selection practically. The challenge which urgently needs to be taken into account is that the precise risk stratification of patients for selecting the specific treatment strategies remains obscure.

The tumor microenvironment (TME) is a mixture that encompasses a comprehensive set of elements such as tumor cells, immune cells, stromal cells, *etc*., nourished by the vasculature (Wu and Dai, 2017; Arneth, 2019). Concerning the importance of TME in cancer development and progression, some researchers have proposed their incisive perspectives that the components infiltrating TME consume the crucial nutrients essential for immune surveillance (Delage et al., 2010; Gross et al., 2014; Klysz et al., 2015), which directly undermine the anti-tumor immunity and indirectly proceed tumor progression. Meanwhile, several studies have documented that the TME characteristics may prevent cytotoxicity T lymphocyte (CTL) and natural killer (NK) cells from recognizing and eliminating tumor cells (Cassetta and Kitamura, 2018; Terry et al., 2019; Bonavita et al., 2020), uncovering the underlying mechanism adopted by the TME in tumor immunomodulation. Although many scientists have shed light on identifying the robust biomarkers or gene signatures characterized by TME features that are extensively associated with the aggressive progression of ccRCC (Chevrier et al., 2017; Mier, 2019; Vuong et al., 2019), few risk stratifications based on TME patterns are available in clinical practice.

This study identified robust TME-related biomarkers significantly associated with ccRCC prognosis and constructed a six-gene signature for risk stratification, which discriminates high- and low-risk groups entitled with different prognoses. In addition, this study demonstrated that the prognostic signature might show indispensable implications on modulating the tumor microenvironment and directing immunotherapy intervention in ccRCC.

## Methods and Materials

### Data Acquisition From The Cancer Genome Atlas

The gene expression profiles of 525 ccRCC samples were extracted from The Cancer Genome Atlas (TCGA) database (https://cancergenome.nih.gov). Samples that met the following criteria were excluded: (1) patients with survival time less than 1 month and (2) patients with incomplete information of TNM, stage, age, gender, and survival time. Then, 468 samples passed the screening and were randomly assigned to the training and validation cohorts by the ratio of 1:1 using random grouping function “sample” in R software. Considering the lack of available and public datasets in clear cell renal cell carcinoma, we hoped to include more samples to validate our findings as much as possible. Therefore, we reset our screening criteria to include the other 51 samples with complete clinical information but with survival time less than 1 month into the validation cohort. As a result, there were 234 samples in the training cohort and 285 samples in the validation cohort. In addition, we included all of the 519 samples into an entire set to further validate our findings.

### Candidate Selection and Gene Signature Construction

The ESTIMATE algorithm could quantify the assessment of the TME characteristics by calculating the immune score, stromal score, and ESTIMATE score (Yoshihara et al., 2013). Only immune score and estimate score passed the preliminary screening, accounting for their high correlations with ccRCC prognosis. A *p*-value <0.05 was considered statistically significant in the log-rank test.

The patients were divided into two groups using the mean value of immune score as the cutoff. We did the same grouping analysis according to the ESTIMATE score. With the help of the “limma” package, the differentially expressed genes (DEGs) of the above-mentioned groups that met the criteria of *p*-value <0.01 and |log_2_ fold change| >1 were subjected to Venn analysis, and 240 overlapping DEGs were filtered out. Kaplan–Meier survival analysis was conducted in the training cohort to find the prognostic DEGs. As a result, 149 prognostic DEGs extracted from the intersection of differential expression analysis and survival analysis were imported into the LASSO Cox regression analysis to prevent data overfitting. The LASSO method is a compression estimate applied for the linear models. It could yield a more refined model by compressing some coefficients, while some coefficients are set to zero. As a result, the valuable variables are filtered out, while the unimportant variables are removed. After conducting the LASSO analysis, only six genes were filtered out with their corresponding coefficients. The detected gene signature was constructed after normalization of gene expression, and the risk score equation weighted by LASSO coefficients was as follows:

$$risk\;score=\sum_{i}Coefficient\left(mRNA_{i}\right)\times Expression\left(mRNA_{i}\right)$$

### Validation of the Prognostic Signature

The risk score of each patient in the entire cohort was calculated according to the above-mentioned formula. Based on the median score, the patients were divided into high- and low-risk score groups in the training and validation cohorts, respectively. Survival analysis and receiver operating characteristics (ROC) curve analysis (Hanley and McNeil, 1982) were performed to evaluate and validate the prognostic value of the gene signature. In addition, univariate and multivariate Cox regression analyses were used to determine whether the risk score was an independent factor from other clinical parameters in predicting ccRCC clinical outcomes.

### Construction and Validation of Nomogram and Decision Tree

The nomogram is a common method widely used in prognostic models. It could integrate diverse prognostic and determinant parameters to predict the probability of an individual clinical event. In this work, we constructed a nomogram based on the clinical variables and risk score extracted from the univariate and multivariate Cox regression analyses to predict the overall survival probability of 1, 3, and 5 years. Then, a calibration curve was plotted to visualize the differences between the nomogram and the actual observed outcomes, while the 45° line represents the best predictability. We also constructed a decision tree to further optimize the risk stratification by removing the redundant elements and highlighting the determinants. After all the decisions, each patient was assigned to one of the branches, and then a more refined risk stratification was generated for personalized decisions.

### Functional Analysis and Consensus Clustering Analysis

The Gene Ontology (GO) and Kyoto Encyclopedia of Genes and Genomes (KEGG) enrichment analyses were performed to identify the enriched pathway DEGs between different risk score groups using the “ggplot2” and “GSVA” packages. We downloaded the latest version of the Hallmark (v7.4) and KEGG (v7.4) gene set collections from the Molecular Signatures Database v7.4 download page (https://www.gsea-msigdb.org/gsea/downloads.jsp), based on which GSEA analysis was implemented using GSEA software (v4.0.3, https://www.gsea-msigdb.org/). Besides this, the Z-score of ssGSEA in the enriched pathway was calculated using the ssGSEA algorithm for normalization (Barbie et al., 2009). According to the optimized *k* value, consensus clustering was used to assign the entire samples into different clusters that might share similar characteristics with the R package “ConsensusClusterPlus” based on the six-gene signature expression profiles (Wilkerson and Hayes, 2010). The clusters revealed significantly different molecular characteristics and survival patterns.

### Correlations Between the Risk Score and Immune-Related Features

The 28 immune cell relevant markers were downloaded from the TISIDB database (http://cis.hku.hk/TISIDB), a user-friendly web portal integrating comprehensive immune-relevant datasets (Ru et al., 2019). The relative proportions of these tumor-infiltrating immune cells were estimated based on the expression levels of these representative markers with the ssGSEA algorithm. In addition, ESTIMATE algorithm was used to quantify the assessment of TME characteristics to investigate the correlations between the risk scores and TME features. We also compared different distributions of the immune checkpoints in the high-risk and the low-risk groups. The correlation coefficients were calculated and graphically displayed in the lollipop diagram and scatterplots.

### Statistical Analysis

All the statistical calculations and visualizations of the results were conducted with R software, version 4.0.3. The Wilcoxon rank-sum test was used to check for the differences between the two independent groups. The Kaplan–Meier plot was performed to show the survival differences of various stratified analyses, and the statistical differences were examined in the log-rank test. Pearson analysis was used to verify the significant correlation coefficients in our study. Unless noted particularly, *p*-value <0.05 was defined as the statistically significant criteria.

## Results

### Identification of TME-Related DEGs

**FIGURE 1:**
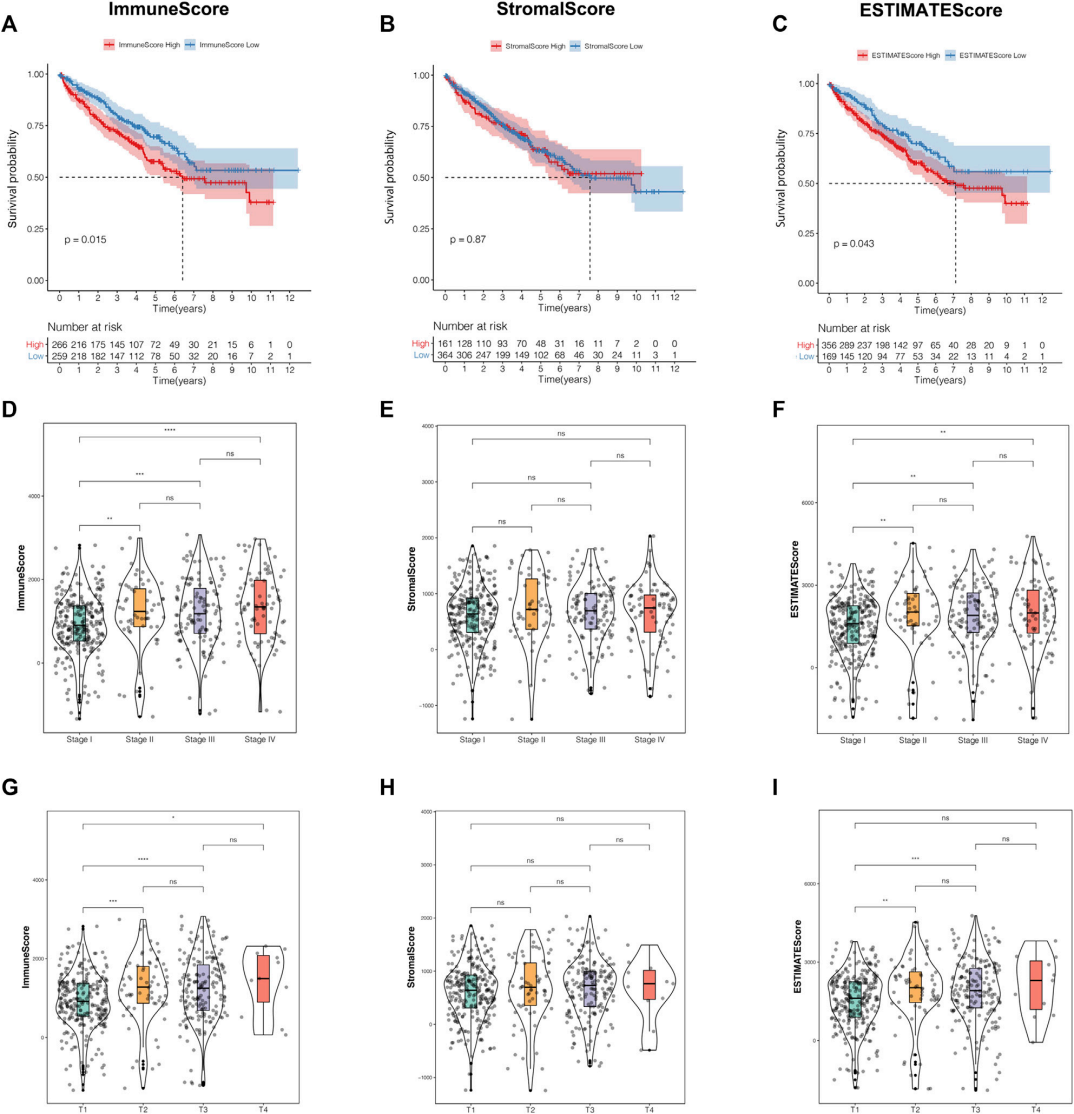
The TME characteristics were correlated with clinical features in ccRCC patients. **(A–C)** The Kaplan–Meier analysis was generated to display the survival significances of different groups stratified by the median value of the immune score **(A)**, stromal score **(B)**, and estimate score **(C)**. **(D–F)** Distribution of the immune score **(D)**, stromal score **(E)**, and estimate score **(F)** in the different stages. **(G**–**I)** Distribution of the immune score **(G)**, stromal score **(H)**, estimate score **(I)** in the different T classifications. TME, tumor microenvironment; ccRCC, clear cell renal cell carcinoma.

The present research was simplified to a flow chart as shown in Supplementary Figure S1. In order to investigate the correlations of the TME characteristics with clinical features in ccRCC patients, we employed ESTIMATE analysis to calculate the TME score using the “estimate” package for each sample in the training cohort. The median value of the TME scores was defined as the cutoff. Kaplan–Meier survival curves revealed that patients with low immune scores or estimate scores encountered a prolonged survival time compared with the others. However, the stratification analysis based on the stromal score made no statistical significance (Figures 1A–C).

Moreover, we observed that the TME scores were closely related to some clinicopathological traits. Apart from the stromal score, immune score, and estimate score, both owned significantly different distributions among various prognosis-related clinical features, such as stage, T classification, and distant metastasis (Figures 1D–I, Supplementary Figures S2A–C). In addition, among the three TME scores, only the estimate score was significantly correlated to lymph node metastasis (Supplementary Figures S2D–F). Unfortunately, there were no significant differences in status stratification among the three TME scores (Supplementary Figures S2G–I). From the perspective of the above-mentioned results, we concluded that immune score and estimate score played a crucial role in ccRCC prognosis, especially in the prediction of survival time and clinicopathological trait discrimination.

### Construction of a Six-Gene-Based Prognostic Signature

**FIGURE 2:**
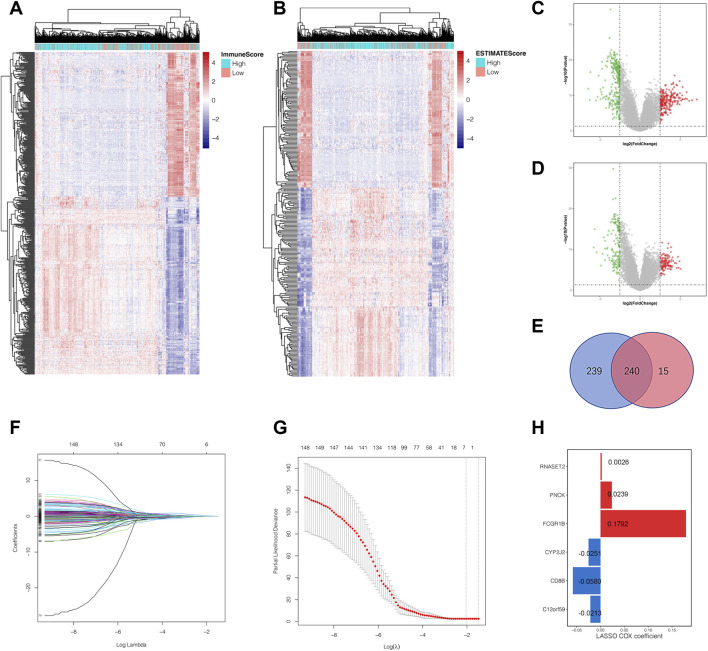
Identification of the overlapping tumor microenvironment-related DEGs and prognostic gene signature construction by LASSO regression analysis. **(A)** Landscape of all DEGs between the high-immune-score group and low-immune-score group. **(B)** Landscape of all DEGs in the high-estimate-score group and low-estimate-score group. **(C)** The volcano plot shows the distribution of DEGs between the high- and low-immune-score groups. **(D)** The volcano plot shows the distribution of DEGs between the high- and low-estimate-score groups. **(E)** The overlapping DEGs were highlighted by the intersection analysis of the Venn diagram. **(F**, **G)** The LASSO regression analysis was employed to identify the most robust prognostic markers for gene signature construction. **(H)** The six genes screened out remained with their individual coefficients. DEGs, differential expressed genes; LASSO, least absolute shrinkage and selection operator.

Based on the median value of immune score and estimate score as cutoffs, we compared the gene expression profiles between different immune and estimate score groups to further explore the underlying mechanism of TME characteristics involved in ccRCC progression. Differentially expressed genes were defined as those that met the criteria of *p*-value <0.01 and | log_2_ fold change| >1 using the R package “limma,” which screened out 479 (Figures 2A,C) and 255 DEGs (Figures 2B,D) from immune and estimate score groups, respectively. As shown in Figure 2E, the Venn diagram displayed 240 overlapping DEGs based on the intersection analysis. Subsequently, Kaplan–Meier analysis was performed to identify 149 common DEGs significantly correlated to overall survival time, which were then imported into LASSO Cox regression analysis in order to prevent overfitting gene signatures (Figures 2F,G). We established a novel prognostic gene signature according to the corresponding coefficients calculated by the LASSO algorithm (Figure 2H). The risk score formula for each sample was constructed as follows based on the expression levels of these mRNAs: risk score = RNASET2*0.0026 + PNCK*0.0239 + FCGR1B*0.1792 + CYP2J2*(−0.0251) + CD8B*(−0.0580) + C12orf59*(−0.0213). After a rigorous calculation of gene expression combined with risk coefficients, the ccRCC samples were then divided into the high- and low-risk score groups using the median risk score value as the cutoff.

### Evaluation and Validation of Six-Gene Signature

**FIGURE 3:**
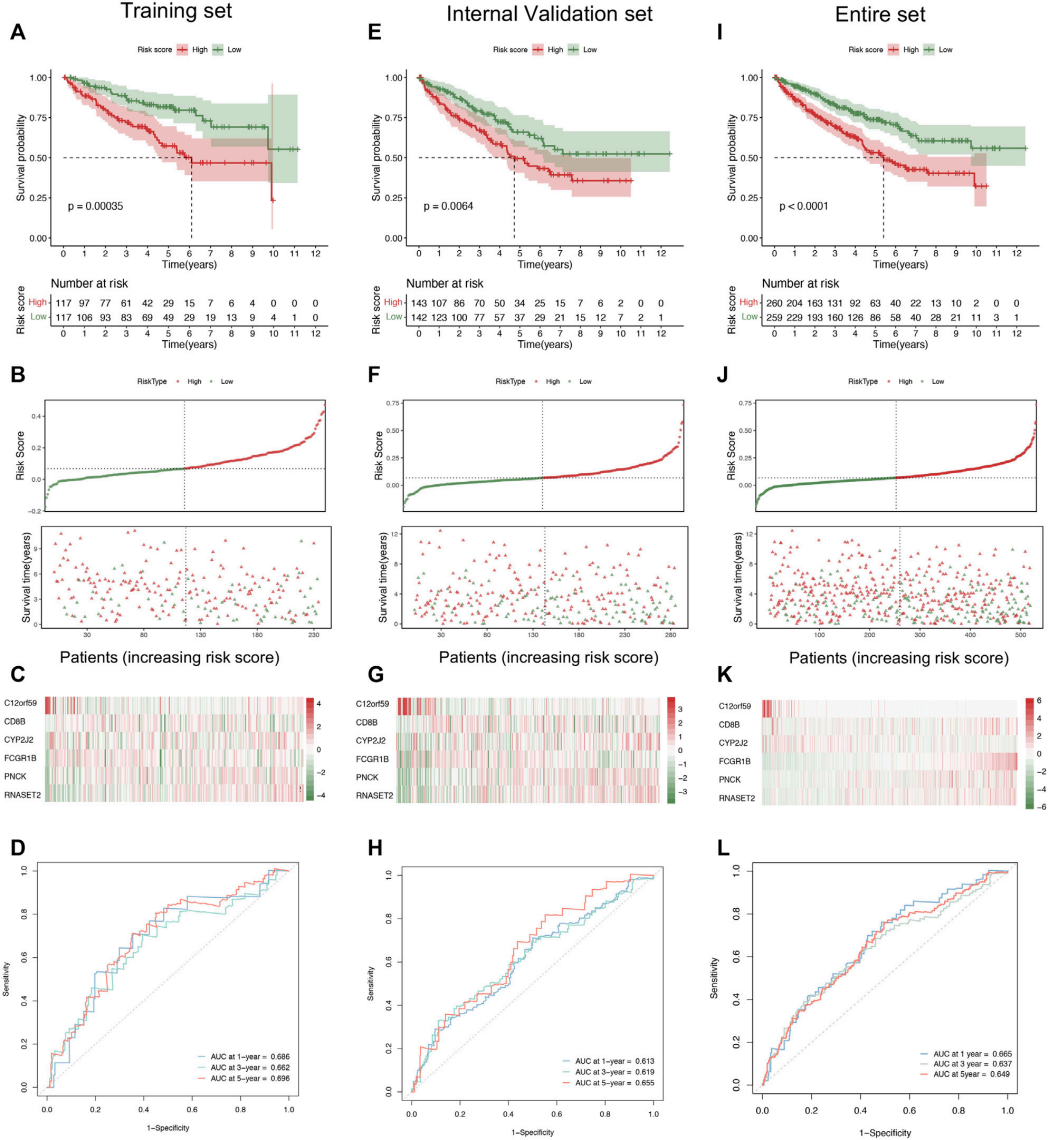
Evaluation and validation of the six-gene signature. **(A)** The Kaplan–Meier analysis based on the risk stratification in the training cohort. **(B**, **C)** The distribution of survival time, patient status **(B)**, and six gene expression profiles **(C)** as the risk score increases in the training cohort. **(D)** Time-dependent ROC analysis represented the accuracy and predictability of the signature in 1-, 3-, and 5-year survival outcome prediction in the training cohort. **(E)** The Kaplan–Meier analysis of the internal validation cohort. **(F**, **G)** The distribution of survival time, patient status **(F)**, and six-gene expression profiles **(G)** as the risk score increases. **(H)** Time-dependent ROC analysis of the internal validation cohort. **(I)** The Kaplan–Meier analysis in the entire cohort. **(J**, **K)** The distribution of survival time, patient status **(J)**, and six-gene expression profiles **(K)** as the risk score increases. **(L)** Time-dependent ROC analysis in the entire cohort. ROC, receiver operating characteristic.

In order to explore the predictive values of the risk score in ccRCC, we conducted several prognosis-related analyses to compare the differences between the high and low groups in the training and validation cohorts, respectively. The Kaplan–Meier survival analysis indicated that the low-risk score group had a prolonged survival time than the high-risk score group (Figure 3A). Notably, the risk score curve and status scatterplot revealed that the deaths mainly accumulated within the high-risk score area in the training cohort (Figure 3B). As shown in Figure 3C, the heat map showed no significant differences between risk scores and the six gene expression profiles, attributing to the limited quantities of the samples. Moreover, time-dependent ROC curves indicated that the area under the ROC curve (AUC) values of 1-, 3-, and 5-year survival were all above 0.65 (Figure 3D), which demonstrated the predictability and the accuracy of the six-gene signature in ccRCC prognosis. To further validate the predictive efficacy of the signature, we conducted the same prognosis-related analyses in the internal validation cohort (Figures 3E–H) and the entire cohort (Figure 3I–L), which turned out to strikingly resemble those in the training cohort. Therefore, we concluded that the six-gene pattern correlated highly to ccRCC prognosis, exhibiting the excellent potential in predicting survival outcomes. As a result, the high-risk score conferred relatively poor clinical outcomes, while the low-risk score conferred a prolonged survival time.

**FIGURE 4:**
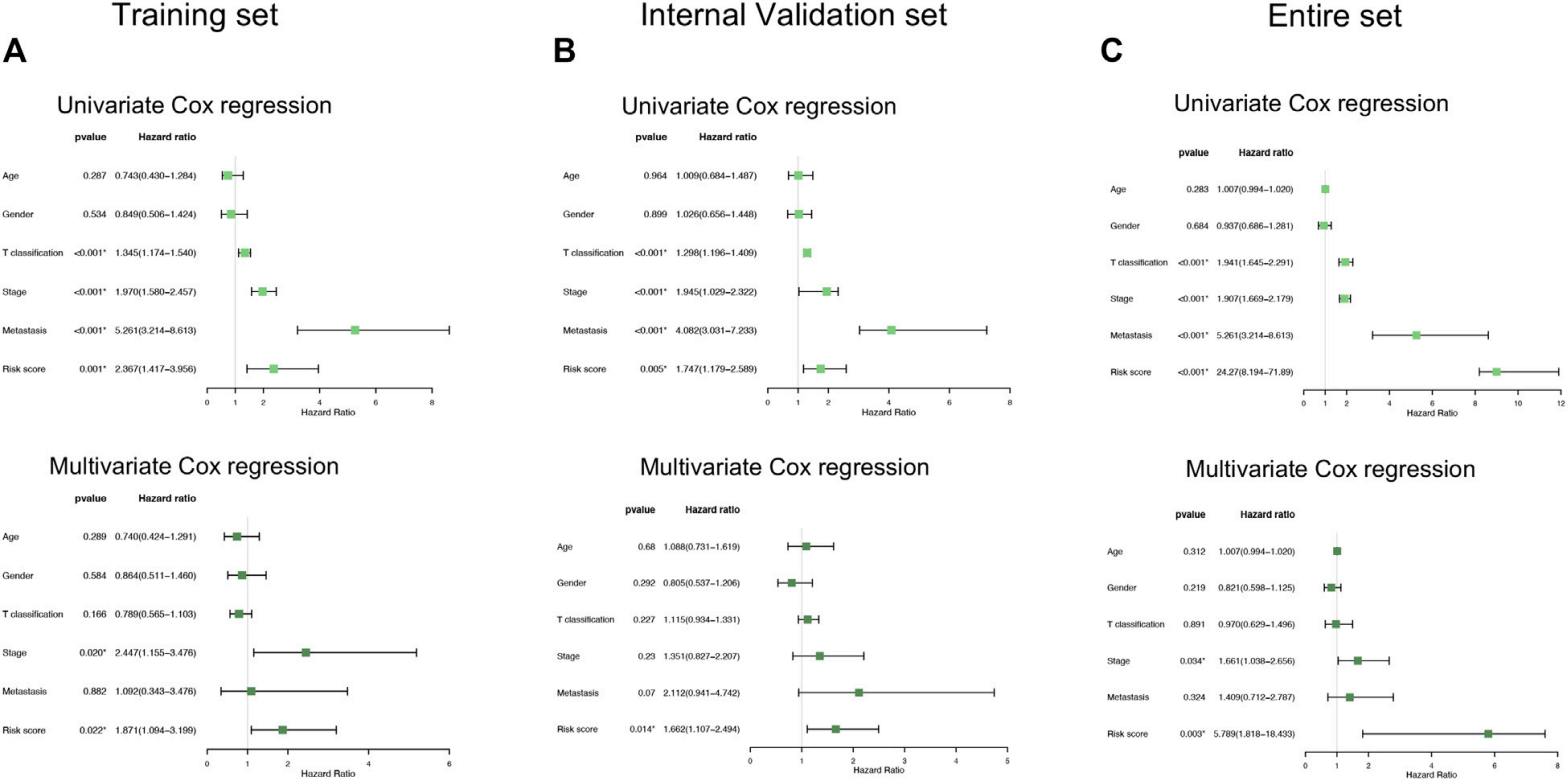
Comparison of the risk score and other clinical parameters in clear cell renal cell carcinoma prognosis. **(A**–**C)** The univariate **(upper)** and multivariate **(lower)** Cox regression analyses were carried out in the training set **(A)**, internal validation set **(B)**, and entire set **(C)**.

We also conducted univariate and multivariate Cox regression analyses in the training and validation cohorts, which focused on several clinicopathological parameters, such as age, gender, T classification, stage, and distant metastasis as well as risk score (Figures 4A–C). From the perspectives of the intersection results, we observed that the risk score stratification might hopefully become a potential and independent factor beyond the other variables concerning the capacity of the prediction in overall survival time.

### The Six-Gene Signature Correlated With the Clinicopathological Traits of ccRCC Patients

**FIGURE 5:**
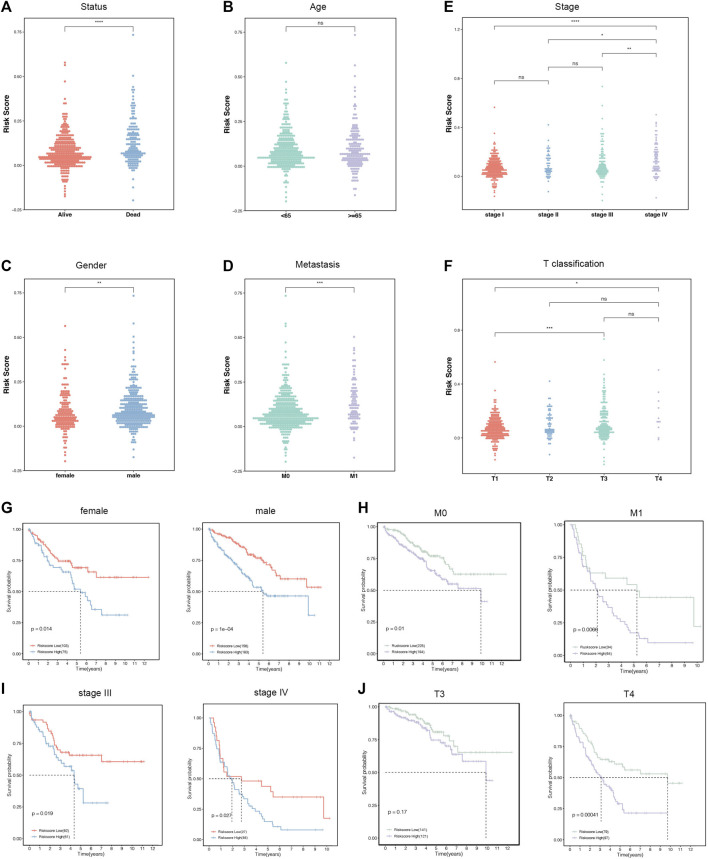
The relationships between the risk score and clinicopathological characteristics. **(A**–**F)** Distribution of the risk score in the stratification analysis based on status **(A)**, age **(B)**, stage **(C)**, gender **(D)**, distant metastasis **(E)**, and T classification **(F)**. **(G**–**J)** Survival analysis showed significant differences between female and male **(G)**, no distant metastasis and distant metastasis **(H)**, stages Ⅲ and Ⅳ **(I)**, and T3 and T4 **(J)**.

The correlations between the six-gene signature and clinical characteristics were further explored in the entire cohorts. The results supported that status, gender, metastasis, stage, and T classification were significantly correlated with the risk scores, while age was not (Figures 5A–F). The high-risk scores indicated more deaths, males, and distant metastasis diseases. In addition, patients divided into later stages and T classification tended to manifest high-risk scores. Subsequently, all the patients were separated into several groups according to clinicopathological features. The Kaplan–Meier survival curves of these groups revealed that the risk stratification represented a good prediction ability in ccRCC prognosis among diverse subgroups. Consistently, the patients with low-risk scores had a better prognosis than those with high scores in female (*p* = 0.014), male (*p* < 0.001), M0 (*p* = 0.01), M1 (*p* = 0.0088), stage III (*p* < 0.019), stage IV (*p* = 0.027), and T4 (*p* < 0.001) subgroups (Figures 5G–J).

### Functional Enrichment and Consensus Clustering Analysis With the Risk Scores

**FIGURE 6:**
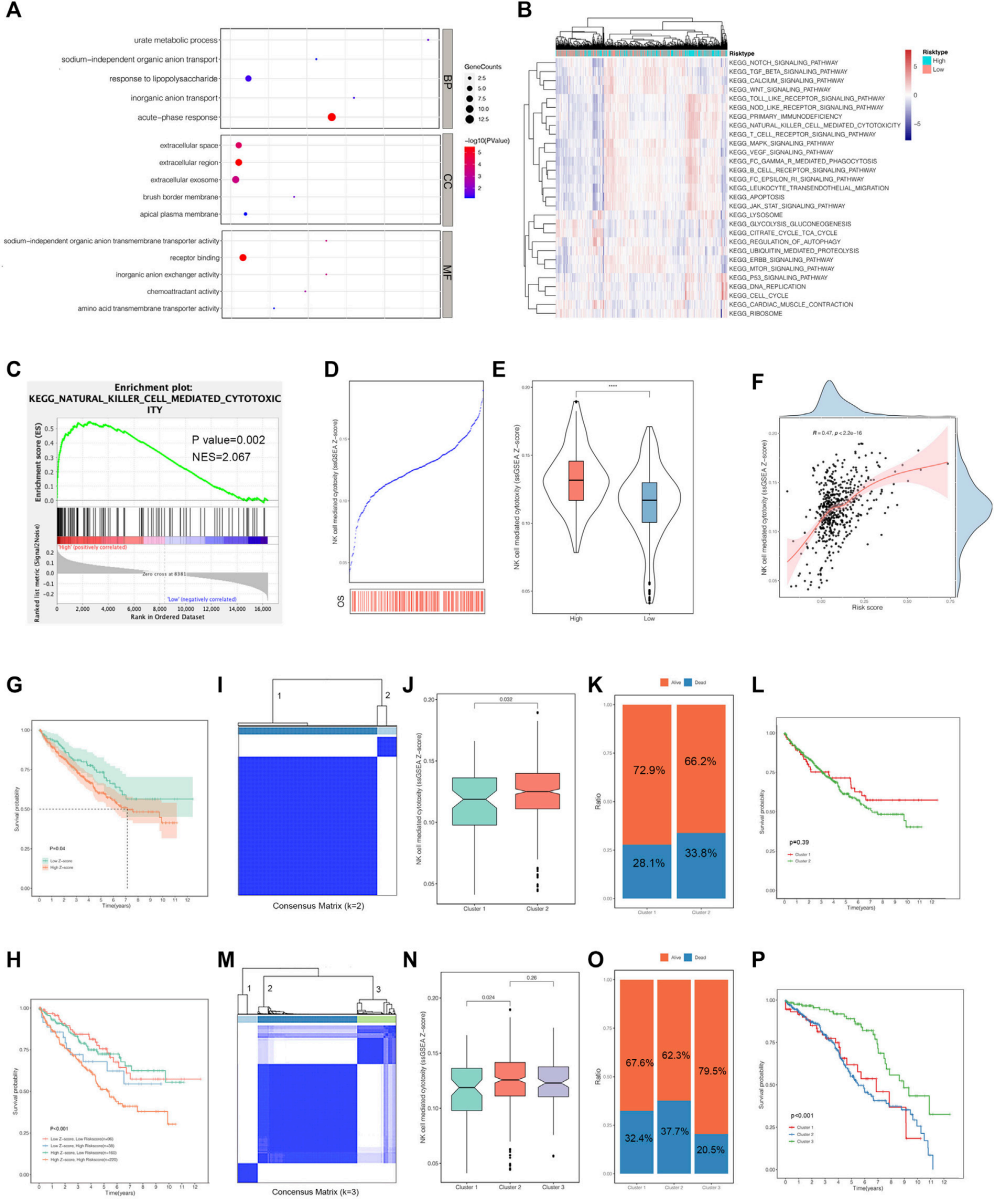
Functional analysis and consensus clustering against the risk score. **(A)** GO annotation of the differentially expressed genes (DEGs) between the high-risk-score group and low-risk-score group. **(B)** KEGG analysis was generated to show the enriched pathways of the DEGs above using the ssGSEA algorithm. **(C)** GSEA indicated the pathway by which NK cell-mediated cytotoxicity was enriched in the high-risk-score group. **(D**, **E)** The Z-score of NK cell-mediated cytotoxicity was positively correlated with the risk score. **(F)** The survival analysis represented the significant differences in the high and low Z-score of NK cell-mediated cytotoxicity groups. **(G)** The combined survival analysis stratified by the risk score and Z-score of NK cell-mediated cytotoxicity. **(I**, **J)** The entire ccRCC samples were divided into two clusters based on the six-gene signature. **(K**, **L)** The survival distribution of the two clusters. **(M**, **N)** The entire ccRCC samples were divided into three clusters based on the six-gene signature. **(O**, **P)** The survival distribution of the three clusters. GO, Gene Ontology; KEGG, Kyoto Encyclopedia of Genes and Genomes; ssGSEA, single-simple Gene Set Enrichment Analysis; GSEA, Gene Set Enrichment Analysis; NK cell, natural killer cell.

Considering the positive correlations between the risk scores and multiple clinical features, we performed GO functional annotation and KEGG enrichment analyses based on the ssGSEA algorithm to quantify the enriched pathway levels of DEGs between high- and low-risk score groups. In view of the results, we observed that acute-phase response was enriched in the biological process term, while extracellular space, extracellular region, and extracellular exosome were in the cellular component term. For molecular function (MF) term, receptor binding was significantly involved (Figure 6A). In addition, the KEGG analysis supported that risk score was positively correlated with immune-related pathways, such as NK cell-mediated cytotoxicity, T cell receptor signaling pathway, leukocyte *trans*-endothelial migration, *etc*. (Figure 6B).

In order to get deep insights into the correlations between risk score and immune-related pathway, we also conducted GSEA analysis based on the risk stratification, which tended to be mutually consistent as previously illustrated. NK cell-mediated cytotoxicity owned significances beyond others (Figure 6C). Subsequently, a comparison of NK cell-mediated cytotoxicity and risk score was established to determine whether the combination could optimize the original model in ccRCC prognosis. The results suggested that the Z-scores of NK cell-mediated cytotoxicity gained in the ssGSEA algorithm were obviously higher in deaths than those alive during follow-up (Figure 6D). Besides this, we confirmed that the Z-scores of the NK cell-mediated cytotoxicity was positively corresponding to the risk scores (Figures 6E,F), inspiring us to perform the survival analysis based on the stratification of the above-mentioned Z-scores. As expected, the high Z-score ones encountered a shorter survival time than the low ones with *p*-value of 0.04 (Figure 6G). In addition, a survival analysis combined with the above-mentioned two components was performed to clarify the intricate relationships among them. The results revealed that the patients with low Z-scores and low-risk scores had a prolonged survival compared to those with low Z-scores and high-risk scores, and patients with high-risk scores were associated with a poorer prognosis than those with low-risk scores based on the same high-Z-score subgroups (Figure 6H). Generally, the risk score could reflect the Z-scores of the NK cell-mediated cytotoxicity. The combined model of the above-mentioned two variables might optimize the original risk stratification for ccRCC prognosis.

Consensus clustering analysis divided the entire samples into different clusters according to the optimal *k* value based on the six-gene signature expression pattern (Figure 6I,M, Supplementary Figure S3). When the *k* value was 2, we observed the particular significances of Z-scores of the NK cell-mediated cytotoxicity and the status distribution between the two clusters (Figures 6J,K). However, as shown in Figure 6L, the survival analysis did not exhibit significant differences. Differently from what was previously explained, when the *k* value was 3, the Z-scores of the NK cell-mediated cytotoxicity of the patients in cluster 1 was significantly different from those in cluster 2 (Figure 6N). The distinct distribution of status stratified by these three clusters is displayed in Figure 6O, and we observed that patients involved in cluster 3 had a superior prognosis to the others (Figure 6P). Given the above-mentioned results, we deduced that the *k* value of 3 manifested pronounced performances in risk stratification beyond the *k* value of 2.

### Correlations of Risk Scores With the Proportion of Tumor-Infiltrating Immune Cells and Immune Checkpoints

**FIGURE 7:**
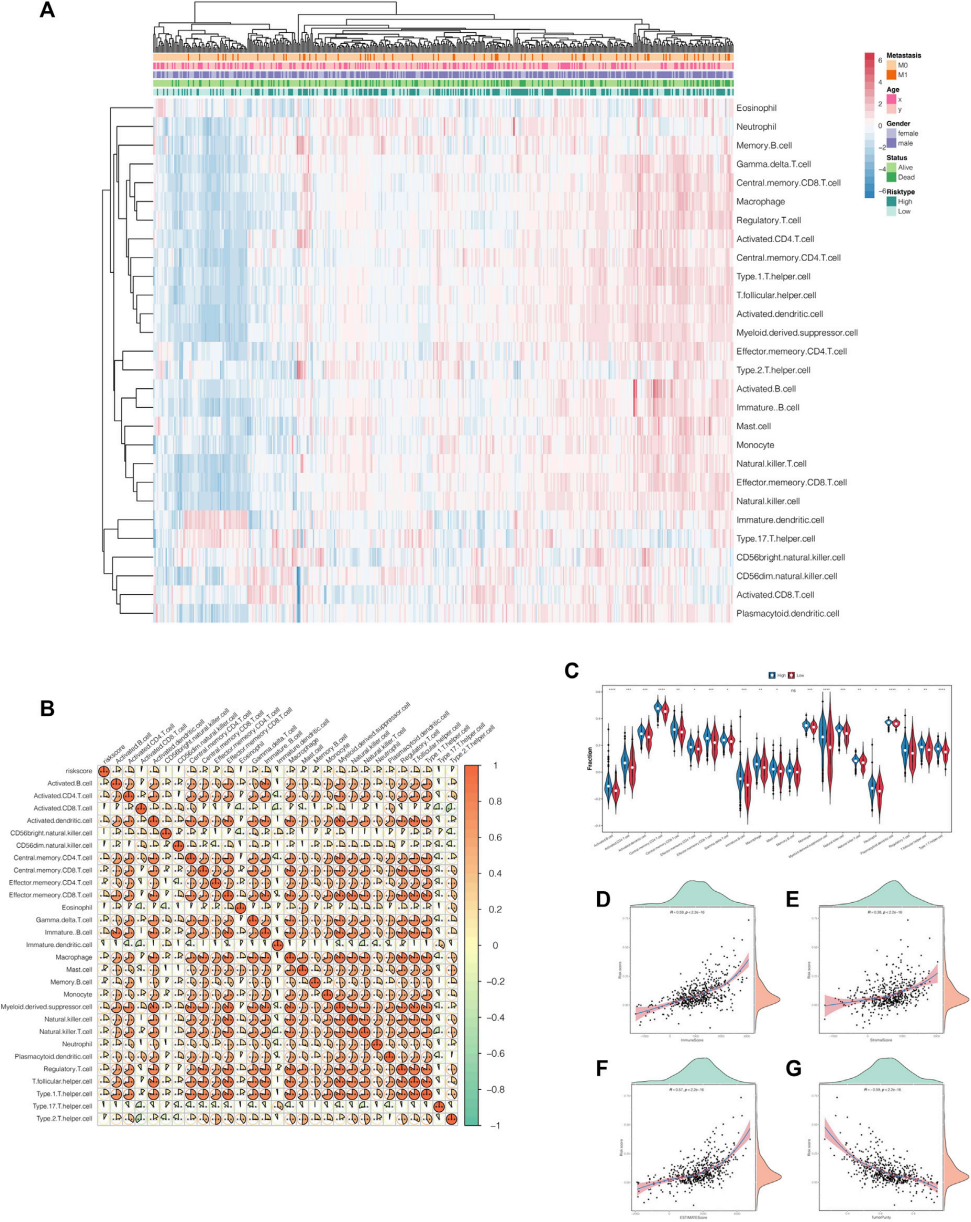
Correlations of the risk scores with the tumor microenvironment characteristics. **(A)** The landscape of the tumor-infiltrating immune cells in the high- and low-risk-score groups. **(B)** Correlation heat map of the risk scores and tumor-infiltrating immune cells. **(C)** The violin plot shows that the tumor-infiltrating immune cells owned higher infiltration levels in the high-risk-score group. **(D**–**G)** The scatterplots combined with the density plots confirmed the positive correlations between the risk scores and immune score **(D)**, stromal score **(E)**, and estimate score **(F)** while negatively correlated with tumor purity **(G)**.

As previously explained, risk scores did correlate with immune-related pathways. Therefore, to further investigate the latent impacts risk scores had on immunity, we extended the ssGSEA algorithm to estimate the proportion of tumor-infiltrating immune cells in ccRCC patients. As shown in Figure 7A, the heat maps represented the landscape wherein most of the immune cells gained higher ssGSEA scores in the high-risk score group, which meant that risk score was positively correlated to immunity activation (Figure 7B). Moreover, the violin plots graphically displayed that high-risk score conferred high immune cell infiltration levels (Figure 7C). We also portrayed the correlations between risk scores and TME characteristics in scatterplots combined with density plots. The results demonstrated that stromal score, immune score, and estimate score were highly and positively associated with risk score, while tumor purity was negatively associated with it (Figures 7D–G).

Interestingly, we compared the expression of immune checkpoints between the high- and low-risk score groups and concluded that the risk stratification might play a crucial role as an indicator of immune checkpoint efficacy, accounting for the positive and significant correlations among them (Supplementary Figure S4).

### Combination of the Gene Signature and Clinical Parameters Improved Risk Stratification and Survival Outcome Prediction

**FIGURE 8:**
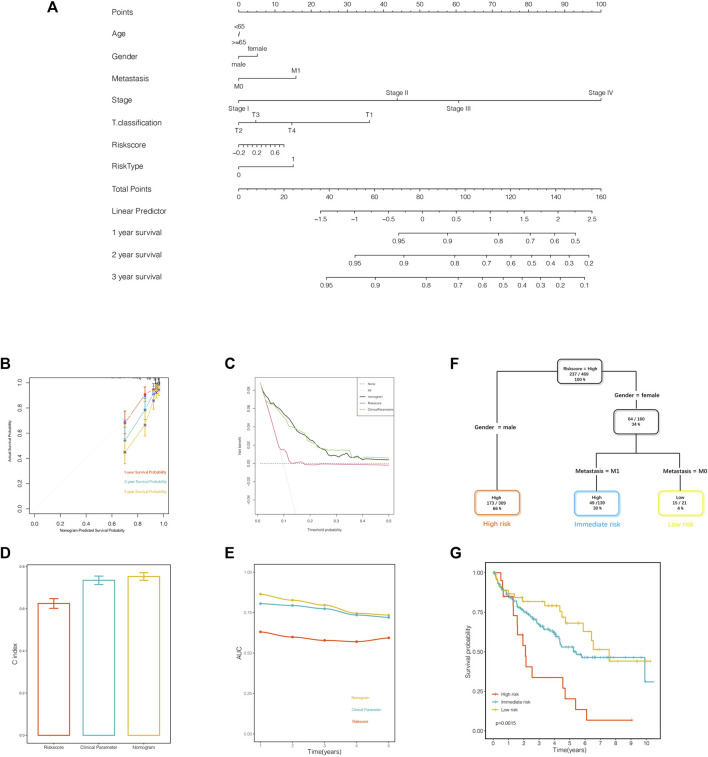
A nomogram and a decision tree were established to improve the risk stratification and predictability of survival outcomes. **(A)** The nomogram was constructed to evaluate the survival probability. **(B)** Calibration analysis indicated the superior predictive probability of 1 year to others. **(C)** Decision curve analysis was generated to display the priority of the nomogram than the variables individually. **(D**, **E)** The C index and area under the receiver operating characteristics (ROC) curve value of the ROC analysis showed that the nomogram owned the best stability and accuracy for survival probability prediction. **(F)** Patients with complete annotation were used to build a decision tree to optimize the risk stratification. **(G)** The Kaplan–Meier analysis confirmed the efficacy and predictability of the optimal subdivisions.

The nomogram was established to quantify the survival probability of an individual clinical event with a risk score, along with other clinical parameters as illustrated previously (Figure 8A). In order to evaluate the efficacy of the nomogram in survival time prediction, we conducted several validation analyses from four distinct perspectives. The prediction line of the 1-year survival ability was practically coincident with the ideal performance compared to 2- or 3-year survival ability in the calibration analysis (Figure 8B), indicating the accuracy of the nomogram in the early period for practical application. As displayed in Figure 8C, the decision curve analysis corroborated that the nomogram, as well as the clinical parameters, obtained much more net benefit of survival probability than the risk score alone. In addition, the C index and AUC value synergistically confirmed that the nomogram provided a superior prognostic value beyond the other variables (Figures 8D,E).

The entire samples with informative clinical annotation and risk score assessment were subjected to establish a decision tree in order to optimize the risk subdivision of overall survival. As shown in Figure 8F, only risk score, gender, and distant metastasis were still retained in the decision tree, and three subgroups were defined according to the above-mentioned three parameters. In the optimized stratification, gender was identified as the cutoff in the high-risk score branch, while distant metastasis replaced gender in the female branch. Interestingly, the survival analysis showed significant differences based on the latest risk subdivision, which was consistent with the survival time prediction (Figure 8G). In summary, both the nomogram system and the decision tree obtained remarkable achievements in optimizing risk stratification and survival outcome prediction, attributing to taking risk score synergistically with clinical parameters into consideration.

## Discussion

To our best knowledge, few studies commit to adopting the risk discrimination of ccRCC related to TME characteristics as a direction in clinical practice, which is exactly the blank our study spares no pain to fill. Based on the high-throughput data and clinical information obtained from the TCGA database, we established a six-gene prognostic signature of pronounced correlations with the TME characteristics with the help of integrated statistical methods. In the training cohort, Kaplan–Meier survival analysis, ROC analysis, and univariate and multivariate Cox analyses were performed to evaluate the predictive capability of the signature in ccRCC prognosis. Subsequently, the dominant findings were confirmed to be repeatable in the validation cohorts using the same statistical methods. In addition, we observed that the signature was distinctly correlated to the infiltration levels of tumor-infiltrating cells and immune checkpoint expression, indicating that the risk stratification might serve as a novel criterion of the management of immunotherapy and patient selection.

After the construction of the prognostic signature, we evaluated the predictability of clinical outcomes based on the stratification of risk scores. We observed that the high-risk zone owned more deaths as well as a shorter survival time. These findings were reproducible in the validation cohorts. Given the above-mentioned results, we concluded that the risk score was negatively correlated to ccRCC prognosis. In addition, the time-dependent ROC analysis confirmed the accuracy and predictability of the signature in long-term prognosis. The univariate and multivariate Cox analysis suggested that the risk score was a pronounced and independent factor of predicting ccRCC outcomes. The corresponding ROC analysis confirmed that the risk score was superior to other clinical parameters. To further investigate the relationships between the risk score and clinical characteristics, we extended the risk score distribution and survival analysis based on different groups stratified by clinical characteristics. The results turned out that the high-risk scores gave rise to more deaths, high-level of clinical-pathological features, and shorter survival time, further verified the findings as previously illustrated.

In order to explore the underlying mechanism that the signature adopted to modulate cancer development and progression, we performed functional analysis to compare the enriched pathways of DEGs derived from high- and low-risk score groups and observed that immune-related and carcinogenetic pathways stand out due to their pronounced performances. Natural killer (NK) cell is generally acknowledged to commit to induce immunosurveillance and hamper tumor aggressive progression through apoptosis activation (Yang et al., 2017; Prager and Watzl, 2019; Sordo-Bahamonde et al., 2020). A previous study agreed that a combined strategy of re-active NK cells and other conventional therapies might give rise to an ideal curative effect for lung cancer patients (Pockley et al., 2020). In the past few decades, several immune-targeted agents for breast cancer that trigger NK cell mediated cytotoxicity have been tested in clinical trials or cell lines (Collins et al., 2012; Juliá et al., 2018). Therefore, we constructed correlation analysis, and survival analysis against NK cell mediated cytotoxicity and observed that it was positively correlated with the risk score. The high Z-score of NK cell mediated cytotoxicity conferred to poor prognosis. Besides, the combination survival analysis of risk score and NK cell mediated cytotoxicity revealed that patients with low levels of these two characters encountered the shortest survival time compared to the others. Taking these findings into account, we supposed that patients assigned to high-risk score groups synergistically with high Z-scores might show greater response on the corresponding NK cell-targeted agents, indicating a novel immunotherapy prospect of ccRCC. Based on the widespread of consensus clustering used in genetic researches recently (Cancer Genome Atlas Resea, 2011; Cancer Genome Atlas Resea, 2012; Cancer Genome Atlas Research Network et al., 2013), we also conducted consensus clustering analysis to optimize the subdivisions for better class discovery (Monti et al., 2003), whose efficacy in prognosis prediction was substantiated in the follow-up survival analysis. Liu et al. considered that tumor-infiltrating immune cells play crucial roles in clinical outcomes prediction and immunotherapy efficacy of lung cancer (Liu et al., 2017). According to another study, kidney cancer expressing high levels of TIM3 separate into two groups in regard to CD8 T-cell infiltration, which may show different implications on immunotherapy targeted TIM3 (Li et al., 2016). The last decades have witnessed great advances taken in tumor-targeted therapy, especially targeting dendric cells (DCs), known as the dominants in the TME that hinder tumor progression (Banchereau and Steinman, 1998; Steinman and Banchereau, 2007; Tran Janco et al., 2015). The correlation analysis in our study revealed that high-risk score conferred to high levels of the majority of tumor-infiltrating immune cells, indicating that high-risk score groups might exhibit activated immune cells-targeted therapy response. Several studies have demonstrated that tumors exhausted DCs through inducing PD-1 expression in order for immune evasion, which can be reversed unless blockade of PD-1 (Krempski et al., 2011; Karyampudi et al., 2014; Dammeijer et al., 2020). In addition, the mechanism that the interaction of tumor-infiltrating lymphocytes expressing PD-1 and PD-L1 undermine antitumor immunity is generally adopted by cancer immunotherapy (Wang et al., 2014; Kurozumi et al., 2017). Tu et al. observed that PD-1 expression was significantly related to several immune cells in many malignancies (Tu et al., 2020). Considering the positively pairwise correlations between tumor-infiltrating immune cells and risk score, PD-1 expression and tumor-infiltrating immune cells, it was reasonable to assume that risk score might share unknown correlations with PD-1. Therefore, the corresponding results supported that many common immune checkpoints gained high expression levels in the high-risk score group, as well as the positive correlation coefficients against the risk score. In the view of the above, we concluded that this risk stratification owned the potential to broaden a new landscape of evaluating immune checkpoint inhibitors response and implementing immunotherapy intervention. Benefited from the nomogram and decision tree, the current six-gene signature was optimized and merged with clinical parameters in order to improve the predictability of ccRCC outcomes. The assessment from four different aspects confirmed the superiority of the combined model compared with the original signature and clinical parameters alone (Xiong et al., 2018; Wang et al., 2020). Besides this, the decision tree made the subdivision based on the specific clinical parameters, which separated the entire cohorts into three subgroups: high-risk group, median-risk group, and low-risk group (Sun et al., 2020; Shi et al., 2021). The subsequent survival analysis revealed the significant differences among the three subgroups, substantiating the necessity of the improved risk stratification.

Generally, in this study, we constructed a six-gene signature, comprehensively evaluated its prognostic values, and correlated this signature with TME-related characteristics among 519 ccRCC samples using an ensemble of integrated analyses. The dominant findings lay the important foundation for optimizing the risk stratification in ccRCC prognosis and understanding the complex intersection relationships with TME modulation. Hopefully, this signature might broaden our cognitions of TME characteristics entitled with tumor progression and propose a new direction in immunotherapy surveillance. Pertaining to the limited data obtained from the TCGA database, these findings need to be further corroborated in a larger cohort or in cytological experiments.

## Data Availability

The original contributions presented in the study are included in the article/Supplementary Material. Further inquiries can be directed to the corresponding author.
