# Supplementary material for: Identification of a Six-Gene Prognostic Signature Characterized by Tumor Microenvironment Immune Profiles in Clear Cell Renal Cell Carcinoma
Source: Front Genet. 2021 Nov 18;12:722421. doi: 10.3389/fgene.2021.722421 (PMC8637193; doi:10.3389/fgene.2021.722421)
Supplement: Supplementary file 1 [file DataSheet1.docx]

**Supplementary Information**


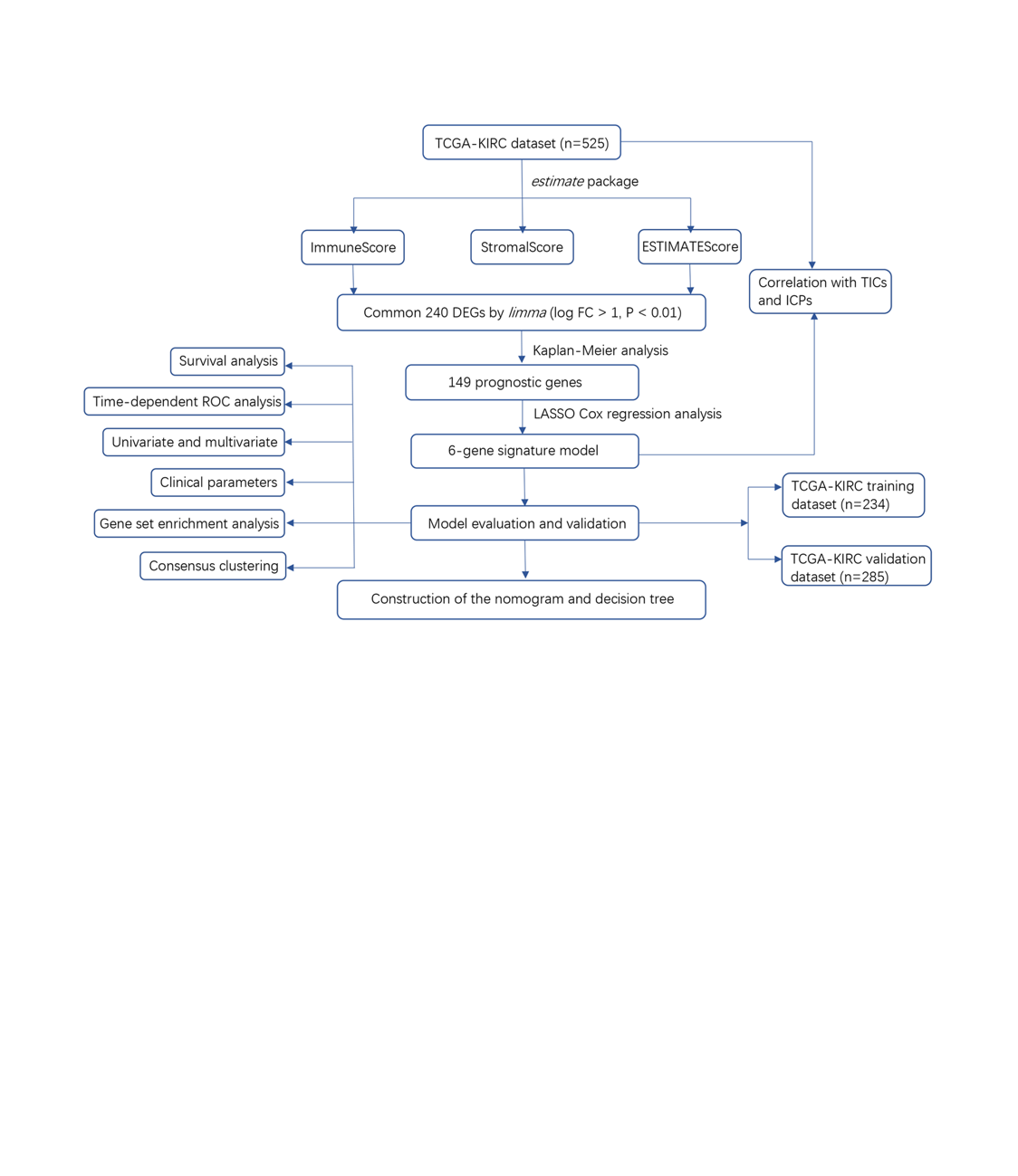


**Figure S1**| Brief flow chart of the study design.


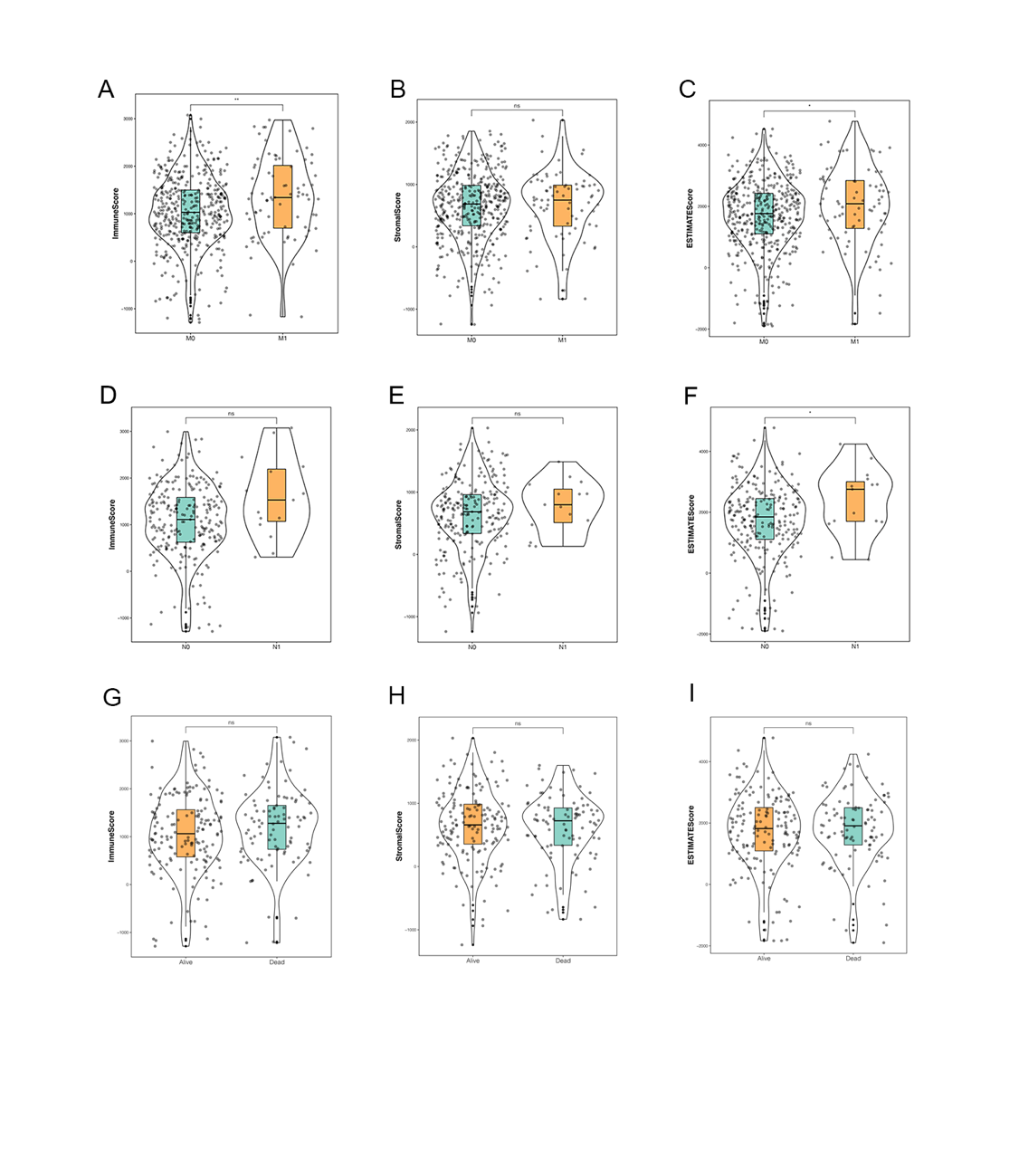


**Figure S2|** The relationships between the risk score and other clinical characteristics. (A-C) The distribution of the immune score (A), stromal score (B), and estimate score (C) in the groups stratified by distant metastasis. (D-F) The distribution of the immune score (D), stromal score (E), and estimate score (F) in the groups stratified by lymph nodes metastasis. (G-I) The distribution of the immune score (G), stromal score (H), and estimate score (I) in the groups stratified by status.


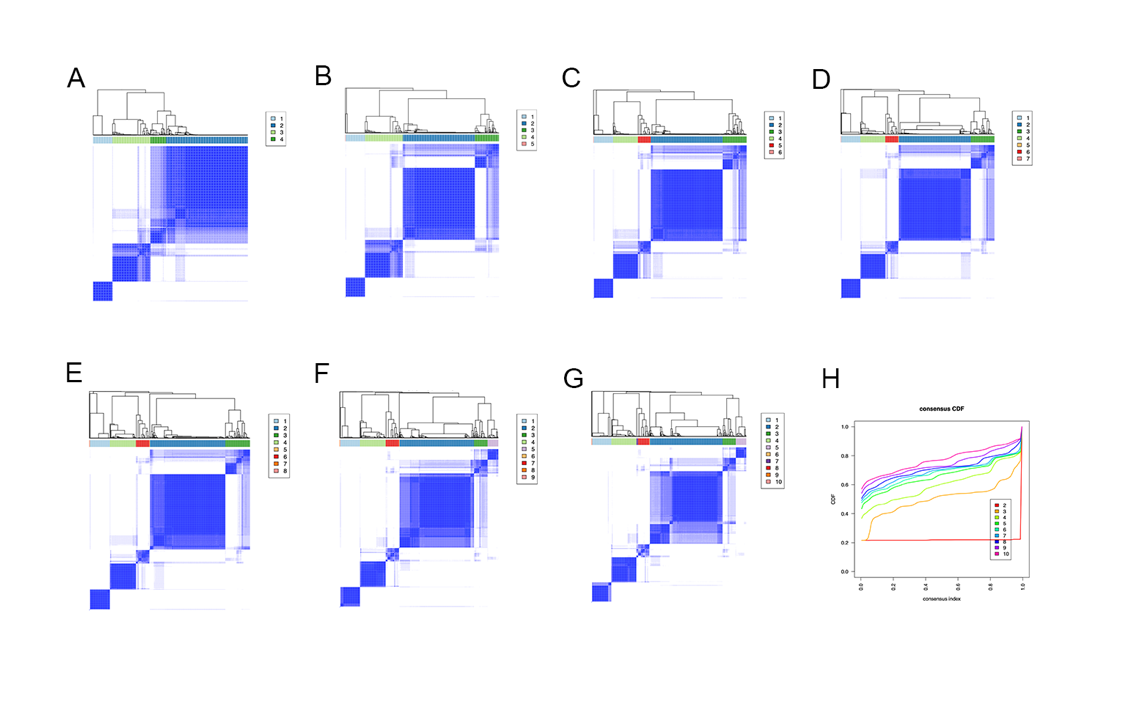


**Figure S3**| Consensus clustering analysis for cluster k=4 to k= 10 of all the ccRCC samples.


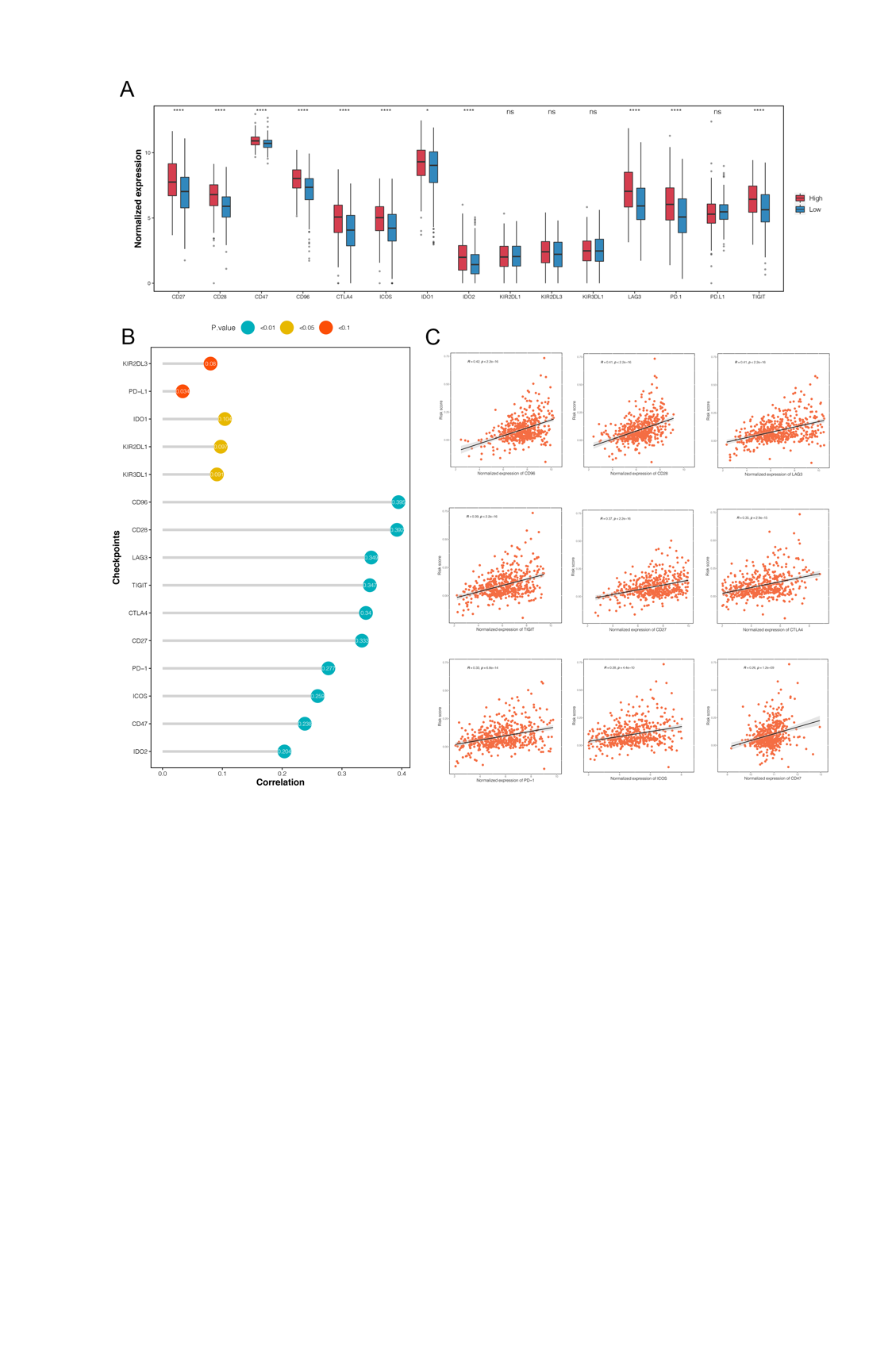


**Figure S4|** Comparison between the risk score and immune checkpoints expression. (A) The boxplot showed that the risk score was highly and positively correlated with immune checkpoints expression. (B) The corresponding coefficients were displayed in the lollipop diagram. (C) The scatterplots combined with fitted line confirmed the highly correlations between these variables.
